# Circulating extracellular vesicles in healthy and pathological pregnancies: A scoping review of methodology, rigour and results

**DOI:** 10.1002/jev2.12377

**Published:** 2023-11-16

**Authors:** Megan V. C. Barnes, Paschalia Pantazi, Beth Holder

**Affiliations:** ^1^ Institute of Reproductive and Developmental Biology, Department of Metabolism Digestion and Reproduction, Imperial College London London UK

**Keywords:** biomarkers, blood, exosomes, extracellular vesicles, microvesicles, placenta, plasma, pre‐eclampsia, pregnancy, serum

## Abstract

Extracellular vesicles (EVs) play a crucial role in pregnancy, revealed by the presence of placental‐derived EVs in maternal blood, their *in vitro* functionality, and their altered cargo in pregnancy pathologies. These EVs are thought to be involved in the development of pregnancy pathologies, such as pre‐eclampsia, pre‐term birth, and fetal growth restriction, and have been suggested as a source of biomarkers for gestational diseases. However, to accurately interpret their function and biomarker potential, it is necessary to critically evaluate the EV isolation and characterization methodologies used in pregnant cohorts. In this systematic scoping review, we collated the results from 152 studies that have investigated EVs in the blood of pregnant women, and provide a detailed analysis of the EV isolation and characterization methodologies used. Our findings indicate an overall increase in EV concentrations in pregnant compared to non‐pregnant individuals, an increased EV count as gestation progresses, and an increased EV count in some pregnancy pathologies. We highlight the need for improved standardization of methodology, greater focus on gestational changes in EV concentrations, and further investigations into the functionality of EVs. Our review suggests that EVs hold great promise as diagnostic and translational tools for gestational diseases. However, to fully realize their potential, it is crucial to improve the standardization and reliability of EV isolation and characterization methodologies, and to gain a better understanding of their functional roles in pregnancy pathologies.

## 1 INTRODUCTION

Extracellular vesicles (EVs) have been implicated in various processes across the course of pregnancy (Mitchell et al., 2015). Initially, EVs may be involved in the attachment and implantation of the blastocyst to the endometrium (Andronico et al., 2019). During pregnancy, EVs may mediate cross‐talk between the mother and the feto‐placental unit, with placental EVs detected in the maternal circulation (Knight et al., 1998) and potentially modulating immune‐ and endothelial cell function (Jia et al., 2018; Lok et al., 2007; Pap et al., 2008) and maternal EVs being taken up by the placenta and modulating placental function (Holder et al., 2016; Rice et al., 2018). Finally, at the end of pregnancy, EVs may initiate or perpetuate inflammatory signals contributing to parturition (Menon & Shahin, 2021).

Given it's positioning between the maternal and fetal circulations, and its central role in establishing and maintaining healthy pregnancy, EVs released by the placenta have been intensely studied in pregnancy. Placental EVs were first detected in maternal blood by Knight et al. (1998), can be detected as early as 6 weeks gestation (Salomon & Rice, 2017), and contain placental‐origin proteins (Holder et al., 2012; Tolosa et al., 2012) and miRNAs (Li et al., 2020; Sadovsky et al., 2015). Circulating placenta‐derived EVs are thought to be mainly produced by the syncytiotrophoblast, the placental epithelial‐like cell layer that is in direct contact with maternal blood (Dragovic et al., 2015; Salomon et al., 2014). Hence, the most commonly utilised marker to detect placental EVs is placental alkaline phosphatase (PLAP), which is highly abundant in the syncytiotrophoblast. Other potential placental markers include syncytin‐1 (Holder et al., 2012; Tolosa et al., 2012) or the chromosome 19 microRNA cluster C19MC (Xie et al., 2014). It is important to note however, that non‐placental EVs may also present useful novel biomarkers in pregnancy pathologies, and this is relatively understudied.

Pregnancy pathologies such as preeclampsia (PE), fetal growth restriction (FGR) and pre‐term birth (PTB) are all significant contributors to maternal and fetal mortality and morbidity, and early prediction of these diseases are desperately needed to assist in the implementation of preventative and therapeutic strategies. Circulating extracellular vesicles, of both placental and non‐placental origin, are an increasingly appreciated source of novel biomarkers for such prediction (Menon et al., 2020; Nair et al., 2018; Tan et al., 2017). However, due to the complexity of blood, the overlapping properties of different entities, and the strong effect of post‐processing procedures on EVs, EV isolation from this biofluid is fraught with difficulties. There is no standardised procedure for EV isolation from blood products, and considerable variation between sites exists, hampering their development as biomarkers. The Blood Task Force, part of the International Society for Extracellular Vesicles Rigor and Standardization subcommittee, plans to set out a roadmap for such standards, based on information provided by the research community (Clayton et al., 2019). Compilation and critical review of discipline‐specific studies that have isolated and/or studied blood‐derived EVs will aid this endeavour.

In this systematic scoping review, we analysed all studies that have isolated and/or characterised circulating EVs in blood from pregnant individuals. We reviewed the methodology of each study, utilising the EV‐TRACK platform (Van Deun et al., 2017), and found significant heterogeneity between studies and areas of low reporting with respect to sample processing and vesicle preparation protocols. This likely contributes to the wide inter‐study variability we observed in EV concentrations. Our comparison of all published studies confirms higher EV concentrations in the blood of pregnant participants compared to non‐pregnant individuals, and a further increase in some pregnancy complications. However, we report high variability in the commonly‐stated increase of EVs in pre‐eclampsia, with some studies measuring a decrease in concentrations compared to healthy controls. We reinforce the importance of detailed methodological reporting and the need for rigor and standardisation in EV isolation and characterisation in studies of circulating EVs in pregnancy.

## 2 MATERIALS AND METHODS

### 2.1 Databases and search strategy

A literature search was conducted on 10th October 2022 through PubMed and MEDLINE (via Ovid), with access to articles granted by Imperial College London subscriptions. The search strategy used to identify all studies of circulating extracellular vesicles in pregnant humans was as follows: ((plasma[Title/Abstract]) OR (serum[Title/Abstract]) OR (blood[Title/Abstract]) OR (circulat*[Title/Abstract])) AND ((extracellular vesicles[Title/Abstract]) OR (exosom*[Title/Abstract]) OR (microvesicles[Title/Abstract]) OR (microparticles[Title/Abstract]) OR (circulating microparticles[Title/Abstract]) OR (STBM[Title/Abstract]) OR (syncytiotrophoblast microparticles[Title/Abstract])) AND (((pregnancy[Title/Abstract]) OR (pregnant[Title/Abstract]) OR (gestational[Title/Abstract]) OR (pre‐eclampsia[Title/Abstract]) OR (preeclampsia[Title/Abstract]) OR (pre‐term[Title/Abstract]) OR (preterm[Title/Abstract]) OR (intrauterine growth restriction[Title/Abstract]) OR (fetal growth restriction[Title/Abstract]) OR ((diabetes[Title/Abstract]) AND (pregnancy[Title/Abstract])) OR ((obesity[Title/Abstract]) AND (pregnancy[Title/Abstract])) OR ((hypertension[Title/Abstract]) AND (pregnancy[Title/Abstract]))). An advanced search was also performed for current clinical trials (*Home—ClinicalTrials.gov*, 2020), which did not produce any results.

Studies that isolated EVs from blood samples (whole blood, plasma, or serum) from pregnant women at any gestation, followed by any type of characterisation and/or quantification of vesicles and/or their surface markers were included, as well as studies that profiled EVs without any isolation steps. Exclusion criteria: EVs were only obtained from *in vitro* cultures, studies of animal models, no primary research data, no EV analysis following isolation, articles not available in English. Screening was performed using Covidence. Each stage of screening was done by MB and PP, with any conflicts being resolved together with BH. All studies were assessed based on their quality of reporting, bias in participant selection, result presentation or author conflict of interest.

### 2.2 Study analysis by EV‐TRACK

All studies were checked against the EV‐TRACK database (https://evtrack.org) (Van Deun et al., 2017), an interactive knowledge base set up for the collation of EV‐based research and the standardisation of quality controlling of methodology. Submitted studies were checked by EV‐TRACK administrators and were assigned a score based on the detail of their methodology and reporting under nine parameters: EV‐enriched proteins, non EV‐enriched proteins, qualitative and quantitative analysis, electron microscopy images, density gradient, EV density, ultracentrifugation specifics, antibody specifics and lysate preparation. Studies that were not already registered were submitted to the database by MB using information present in the manuscripts.

### 2.3 Data extraction and statistical analysis

Patient data was collated, including gestational age, diagnosis of gestational pathology and inclusion of healthy or non‐pregnant controls. Data relevant to EV analysis, including sample type, methodology of EV isolation and characterisation, were recorded. Any quantitative and qualitative results were also extracted. A heterogeneity plot was produced in RStudio (v4.1.1), using packages ggplot2 (v3.4.0), tidyverse (v1.3.2) and reshape (v1.4.4). Where applicable, a Shapiro–Wilk test was used to test for normality of data distribution, and data in all groups tested were skewed (alpha ≠ 0.05, *P* > 0.05). For comparison of two groups, the Wilcoxon test was used for paired analyses, and the Mann–Whitney *U* test for unpaired analyses. For comparison between more than two groups, the Friedman test followed by a Dunn's post‐hoc was employed for paired, and the Kruskal–Wallis for unrelated samples. Where stated, fold‐changes were calculated and Log_2_ transformed and tested using a One‐sample Wilcoxon test. *P* values of <0.05 were deemed statistically significant. Statistical analyses were performed using GraphPad Prism (v9.1.0).

## 3 RESULTS

### 3.1 Studies of circulating EVs in pregnancy

**FIGURE 1:**
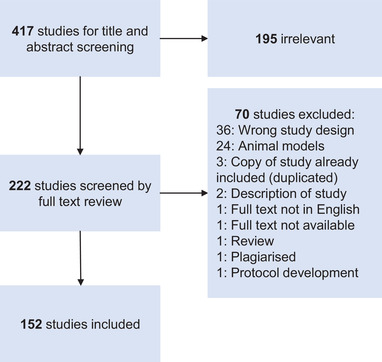
Summary of study selection for systematic review and meta‐analyses (PRISMA).

**FIGURE 2:**
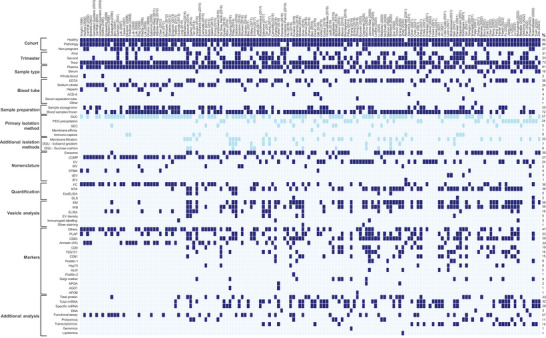
Methodology heterogeneity in studies of circulating EVs in pregnancy. The 152 included studies ordered by publication year, reporting EV isolation, quantification and analysis methods. For EV isolation, the light blue colouration indicates when a combination of methods was employed. The percentage of studies that reported a specific parameter, from each category, is shown on the right. ACD‐A: anticoagulant citrate dextrose solution, AGO1: argonaut RISC component‐1, ALIX: ALG‐2 interacting protein X, APOA/B: apolipoprotein A/B, (C)MP: (circulating) microparticle, DGU: density gradient ultracentrifugation, DLS: dynamic light scattering, DUC: Differential ultracentrifugation, EDTA: ethylenediaminetetraacetic acid, ELISA: enzyme‐linked immunosorbent assay, EM: electron microscopy, EV: extracellular vesicle, FC: flow cytometry, Hsp70: heat‐shock protein 70, lEV: large EV, MV: microvesicle, NTA: nanoparticle tracking analysis, SEC: size‐exclusion chromatography, STBM: syncytiotrophoblast microparticle, WB: western blot.

Following duplicate exclusion, a total of 417 studies were identified and screened by their title and abstract, with 195 then excluded based on the pre‐defined inclusion/exclusion criteria. One additional study (DOI: 10.26355/eurrev_201804_14733) was excluded due to plagiarism (reported to the journal, retraction notice published: DOI: 10.26355/eurrev_202206_28944). A final, full‐text screening of the remaining 222 excluded a further 70 studies, resulting in 152 studies for final inclusion in this scoping review (Figure 1). Although numbers remain modest, between 1998 to 2022, there was a net increase in the number of pregnancy specific EV studies published per year, with 20 studies published in 2022 by the 10th October 2022 (Figure S1). The methodological details of all studies are presented in a heterogeneity plot (Figure 2).

### 3.2 Patient characteristics

All studies, apart from one (Abolbaghaei et al., 2021), studied EVs from the blood of healthy pregnant women, and the majority (86%) also collected samples from women with a complication of pregnancy or co‐morbidity (Figure 2). Circulating EVs have been investigated in a wide range of pregnancy pathologies and co‐morbidities (Table S1), but by far the most studied is pre‐eclampsia (PE) (*n* = 76; 50%), followed by gestational diabetes mellitus (GDM) (*n* = 13) and pre‐term birth (*n* = 12). Just under a third of the studies (27%) included a non‐pregnant comparator group (Figure 2). Most studies sampled in just one trimester (*n* = 111; 73%), but some sampled in two (*n* = 10; 7%) or all three trimesters (*n* = 26; 17%). Most samples were taken in the third trimester (*n* = 111; 73%), followed by the second (*n* = 52; 34%) and the first trimester (*n* = 47; 31%). Five studies did not report the gestational age at blood sampling.

### 3.3 Blood sample collection and processing

**FIGURE 3:**
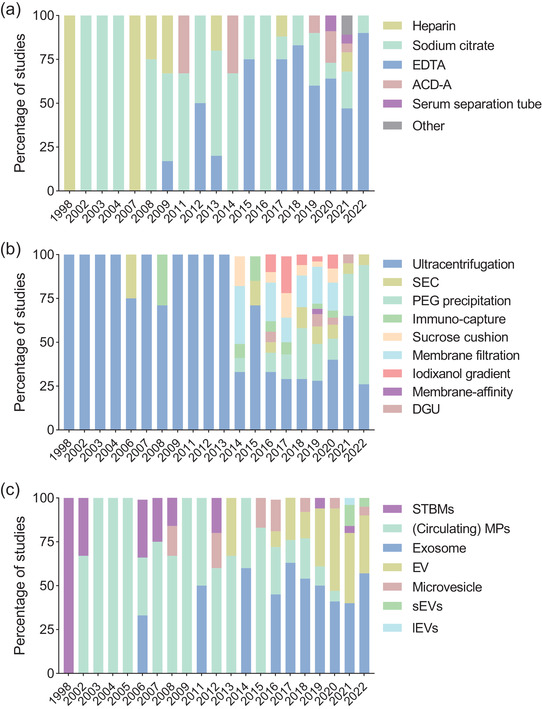
Chronological changes in EV methodology and terminology. (a) Blood tube type sorted by year of publication *(n* = 115). (b) EV isolation method sorted by year of publication. Some studies combined more than one methods, so are represented more than once. (c) EV nomenclature sorted by year of publication. ACD‐A: acid citrate dextrose‐A, DGU: density gradient ultracentrifugation, EDTA: ethylenediaminetetraacetic acid, EV: extracellular vesicle, lEVs: large EVs, PEG: Polyethylene glycol‐based precipitation, SEC: size‐exclusion chromatography, sEVs: small EVs, STBM: syncytiotrophoblast microparticle.

Most studies isolated EVs from plasma (123/152; 81%), with 25 studies (16%) isolating EVs from serum (Figure 2). Four studies performed direct analysis of EVs in whole blood. Blood tube type is summarised in Figure 3a. ‘Other’ refers to two studies which used either a tube without anticoagulant (Cui et al., 2021), or a Streck cfDNA BCT tube (Yaşa et al., 2021). Only six of the 25 serum studies reported the type of blood tube utilised. Of the studies utilising plasma, 89% (135/152) reported the type of blood tube/anti‐coagulant. Notably, heparin appears to be less commonly used after 2009. EDTA has been the most commonly used anti‐coagulant (58/152; 38%), followed by sodium citrate (37/152; 24%), with EDTA becoming particularly utilised from 2015 onwards. About a third (45/123; 37%) of the included studies stated the plasma storage time, and 76% (93/123) reported whether plasma samples were frozen prior to EV isolation (Figure 2). Regarding centrifugation of plasma, important for rapidly removing platelets which can release EVs *ex vivo*, 38 studies (31%) did not specify their protocol; 42 (34%) studies used one centrifugation step between 1500–4000 × *g* and 38 (31%) studies used two centrifugation steps (nine at the same speed, 29 at two different speeds). Four studies (3%) used greater than two centrifugation steps, and one study deliberately analysed platelet‐rich plasma (one 160 × *g* centrifugation step) (Ling et al., 2014). Of the 25 serum studies, 11 (44%) did not specify their centrifugation protocol; 11 studies (44%) used one centrifugation step; one study used two centrifugation steps at different speeds, and one study used more than two centrifugation steps prior to EV isolation.

### 3.4 EV isolation

The methods of EV isolation, quantification, profiling, and further analyses utilised in the 152 studies of circulating EVs in pregnancy are presented as a heterogeneity plot in Figure 2, and chronologically in Figure 3. The most utilised EV isolation method was differential ultracentrifugation (DUC) (86/152; 57%), followed by polyethylene glycol (PEG)‐based precipitation (37/152; 24%) or size exclusion chromatography (SEC) (13/152; 9%). Nineteen studies utilised more than one primary isolation techniques, and 18 studies did not use any isolation technique but went straight to vesicle analysis by flow cytometry or extracted EV‐ associated miRNA. DUC was used on its own in all but three studies before 2014 (95%), after which it dropped from 71% (2015) to 26% of studies (2022), as other methods became more popular, such as SEC or PEG precipitation, or was supplemented with other isolation techniques such as membrane filtration (Figure 3b). PEG precipitation is still used as recently as 2022, despite evidence that it co‐isolates many contaminants from blood, such as high‐ or low‐density lipoproteins (HDL/LDLs) (Brennan et al., 2020). Methods used in combination with DUC included: 0.22 μM membrane filtration (used by 33/152; 22% of studies), iodixanol gradient (7%) or sucrose cushion (6%). Eleven studies (7%) used two of the supplementary isolation techniques, and one study used all three. Combination of isolation techniques was not common before 2014.

### 3.5 EV nomenclature

A range of EV nomenclature has been employed in pregnancy studies, changing with time (Figures 2 and 3c). Across all studies, ‘exosomes’ is the most popular (39%), followed by MPs (27%), EVs (24%), and others (Figure 2). The earliest EV studies in pregnancy used the term ‘microparticles’ (MP) or ‘syncytiotrophoblast microparticles/microvesicles’ (STBM), which was first introduced in 2002 (VanWijk et al., 2002a; VanWijk et al., 2002b). It is important to note that the term STBMs has also been used to refer to ‘syncytiotrophoblast microvillous membranes’ (Smárason et al., 1993), and ‘syncytiotrophoblast microvilli’ (Knight et al., 1998). In this latter study, although they utilise the term ‘syncytiotrophoblast microvilli’, their method of isolation (ultracentrifugation) is the same as that employed for EVs, and so this study was included in this review. The terms MP and STBM remained popular in the field until around 2016 and are still in use by some groups. It was noted that just over a third of studies quantifying the cellular source of the EVs referred to their vesicles as ‘microparticles’ or ‘circulating microparticles’ (MP/cMP) (23/68; 34%). The term ‘exosome’ first appeared in 2006, and overall is the most common term in pregnancy studies (59/152; 36%; Figure 2). However, in recent years the term ‘extracellular vesicle’ (EV), first employed in 2013, has become much more prevalent, particularly from 2019 onwards, making up almost half (47%–33%) of studies published between 2019–2022 (Figure 3c). Finally, four studies (all from 2021/2022) referred to their vesicles as small EVs (sEV), and one study from 2021 used the term large EV (lEV).

The majority of studies that use the term ‘exosome’ did not utilise methodology for specific isolation of exosomes, that is, vesicles that have derived from the endosomal pathway, as recommended in current MISEV guidelines (Théry et al., 2018) (Figure 2). Of note, 21 studies used only commercial PEG precipitation‐based isolation (ExoQuick, Total Exosome Reagent, etc.), which is not specific to any EV subtype, and is particularly subject to contamination with non‐EV components, particularly from blood. The most popular methodology for ‘exosome’ studies was ultracentrifugation, which was used by 31 studies, sometimes alone (*n* = 3), but usually in combination with other methods, most commonly membrane filtration (*n* = 20). This would not result in isolation of pure exosomes, especially from blood. Some studies did use methods that can better enrich for exosomes and other small EVs, such as gradient centrifugation. No ‘exosome’ studies isolated EVs using proposed exosome markers such as tetraspanins.

### 3.6 EV‐TRACK scoring

**FIGURE 4:**
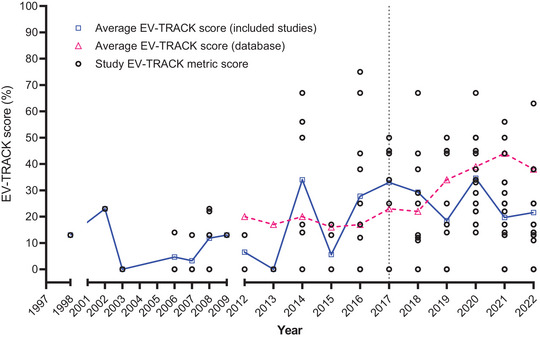
EV‐TRACK analysis of studies on circulating EVs in pregnancy. EV‐TRACK scores assigned to each of the included studies organised by year. The solid blue line indicates the mean (or score for pregnancy studies included in this review, while the magenta dashed line indicates the mean score for all EV studies across the whole EV‐TRACK database (available from 2012 onwards). The vertical dotted line at *x* = 2017 represents the year that EV‐TRACK became publicly available.

Of the 152 identified studies, 128 had isolated EVs prior to analysis and hence were eligible for registration on EV‐TRACK, but only five studies were present when we commenced this review (Dragovic et al., 2013; Salomon et al., 2014; Sarker et al., 2014; Tan et al., 2014; Vargas et al., 2014). We therefore collated and submitted the information from the remaining 123 unregistered studies, and subsequently plotted all 128 studies against their year of publication, compared to the average EV‐TRACK metric scores for all submitted studies over the same period (Figure 4). For most years, pregnancy studies were equal to, or below the average metric of all studies submitted to EV‐TRACK. There was considerable variation between studies, with many still recording scores below 30%, but more studies have scored above 40% since 2014. Analysis of the nine experimental parameters that contribute to EV‐TRACK scores showed that the presence of EV‐enriched proteins was the most reported parameter (61% of studies), and thus was the largest contributor to higher EV‐TRACK scores. Approximately one quarter of relevant studies reported their antibody specifics (28%) and/or qualitative and quantitative analysis (26%). Fewer studies reported the absence of non‐EV‐enriched proteins (17%), ultracentrifugation protocol information (16%), lysate preparation (15%), density gradient information (14%), vesicle density (10%) and electron microscopy images (3%), making these the main contributors to lower EV‐TRACK scores.

### 3.7 Measurement of EV markers/cargo

Sixty‐five (43%) of studies measured EV‐associated proteins (CD81, CD63, CD9, TSG101, Flotillin‐1/2, HSP70 or ALIX), and 16 studies looked for non EV‐associated proteins (or contaminants) (Golgi marker (Grp94), apolipoprotein A/B (APOA/B), protein argonaute‐2 (AGO2)). The most common EV‐associated surface markers measured were the tetraspanins CD63 (*n* = 56; 37%), CD9 (*n* = 29; 19%) and CD81 (*n* = 23; 15%; Figure 2). These surface markers were also measured by fluorescent NTA (fNTA) or flow cytometry (FC) in some studies. Cytosolic protein markers were less commonly included/reported: TSG101 (*n* = 25; 16%), Hsp70 (*n* = 9; 6%), ALIX (*n* = 5; 3%) and Flotillin 1/2 (*n* = 9/1; 6/1%, respectively). Lipoprotein markers (Apolipoprotein A/B, argonaute‐2, etc), as one measure of major contamination of vesicle preparations, were only included in six studies. Thirteen (9%) studies included the Golgi marker Grp94 as a negative control in their WB analysis. Of the 48 studies that measured either total or specific miRNA expression from vesicle preparations, 32 (67%) measured at least one EV‐enriched surface or cytosolic marker.

### 3.8 EV isolation and quantification methodology, and its effect on circulating EV concentrations reported in pregnancy

**FIGURE 5:**
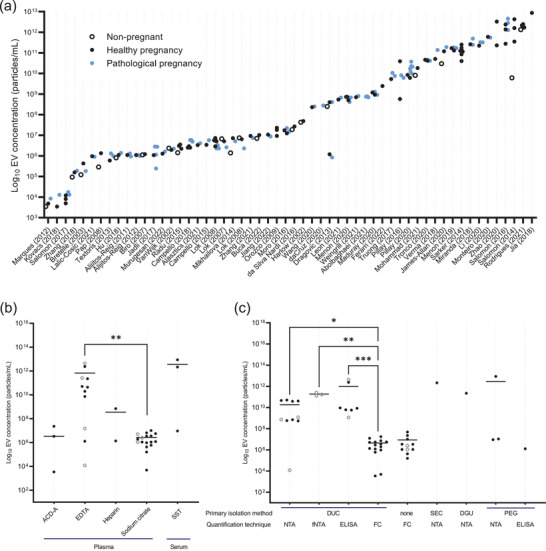
EV concentrations in the blood of healthy pregnancies, pathological pregnancies and non‐pregnant women varies by quantification method. (a) Total EV concentrations measured per ml of plasma/serum plotted on a Log_10_ scale in ascending order. Open circles; non‐pregnant women, black circles; healthy pregnancies, blue circles; pathological pregnancies. (b) Total EV concentration isolated per ml of plasma/serum, in healthy pregnant participants in their third trimester, measured in plasma (*n* = 38) or serum (*n* = 3). EV concentration measurements were compared by Kruskal–Wallis followed by Dunn's post‐hoc test. * *P* < 0.05; ** *P* < 0.01. Filled black circles represent data reported in the paper whilst grey points represent data extrapolated from graphs, black solid lines represent median for each data set. ACD‐A: anticoagulant citrate dextrose solution, EDTA: ethylenediaminetetraacetic acid, SST: serum separation tube. (c) Total EV concentration isolated per ml of plasma/serum, in healthy pregnant participants in their third trimester (*n* = 42), separated by primary isolation method, and method of quantification. EV concentration measurements for the studies that utilised DUC were compared by Kruskal–Wallis followed by Dunn's post‐hoc test. * *P* < 0.05; ** *P* < 0.01. Filled black circles represent data reported in the paper whilst grey points represent data extrapolated from graphs, black solid lines represent median for each data set. DUC: differential ultracentrifugation, DGU: density gradient ultracentrifugation, FC: flow cytometry, NTA: nanoparticle tracking analysis, PEG: Polyethylene glycol‐based precipitation.

Total EV concentrations were measured in 76 studies. Results from 56 of these were included in our analysis as the other twenty studies did not report their vesicle concentrations as /mL or /μL of starting material (Cui et al., 2021; Desprez et al., 2008; Elfeky et al., 2017; Ezrin et al., 2015; Franzago et al., 2021; Freeman et al., 2008; Holthe et al., 2005; Jørgensen et al., 2020; Levine et al., 2020; Meziani et al., 2008; Motawi et al., 2018; Rajaratnam et al., 2021; Sabapatha et al., 2006; Salomon et al., 2009; Sarker et al., 2014; Tan et al., 2017, 2014; Tesse et al., 2007; Uszyński et al., 2013). The results from non‐pregnant, healthy pregnant and pathological pregnant cohorts are summarised in Figure 5a. Reported concentrations of blood EVs ranged from as low as 3.5 × 10^3^ particles/mL (Marques et al., 2012) to as high as 8.7 × 10^12^ particles/mL (Jia et al., 2018). Total EVs quantified by flow cytometry were measured by vesicles expressing phosphatidylserine (PS), which is commonly expressed by EVs and detectable by either Annexin‐V or Lactadherin (Lalic‐Cosic et al., 2021; Wei et al., 2018).

To elucidate any effect of quantification method on measured EV concentrations, studies of healthy pregnancies sampled in the third trimester were further analysed, as this was the largest pregnant dataset (*n* = 42 studies). Within these, six studies presented results on two timepoints within the third trimester; an average of these were used for analysis. Of 42 studies, three used serum, 38 used plasma, and one did not specify. Small numbers precluded comparison between plasma or serum sample types, but within the different plasma anticoagulants, we observed significantly higher EV concentrations when EDTA was utilised compared to sodium citrate (*P* = 0.008) (Figure 5b). When comparing EV quantification methodology, statistical comparison between quantification methods was only performed for studies that utilised differential ultracentrifugation (DUC) for isolation, due to small *n* numbers in other groups. Studies that quantified EVs by NTA, fNTA and ELISA reported significantly higher concentrations than those quantified by flow cytometry (NTA: Log_2_ fold‐change 12, *P* = 0.012; fNTA: Log_2_ fold‐change 15.4; *P* = 0.002; and ELISA: Log_2_ fold‐change 17.7; *P* < 0.001). NTA, fNTA and ELISA all yielded comparable EV concentrations (*P* > 0.05). Vesicle concentrations measured by flow cytometry were consistent between studies that had isolated EVs by DUC, and those that quantified EVs directly from platelet‐free plasma.

### 3.9 Circulating EV concentrations in pregnancy

**FIGURE 6:**
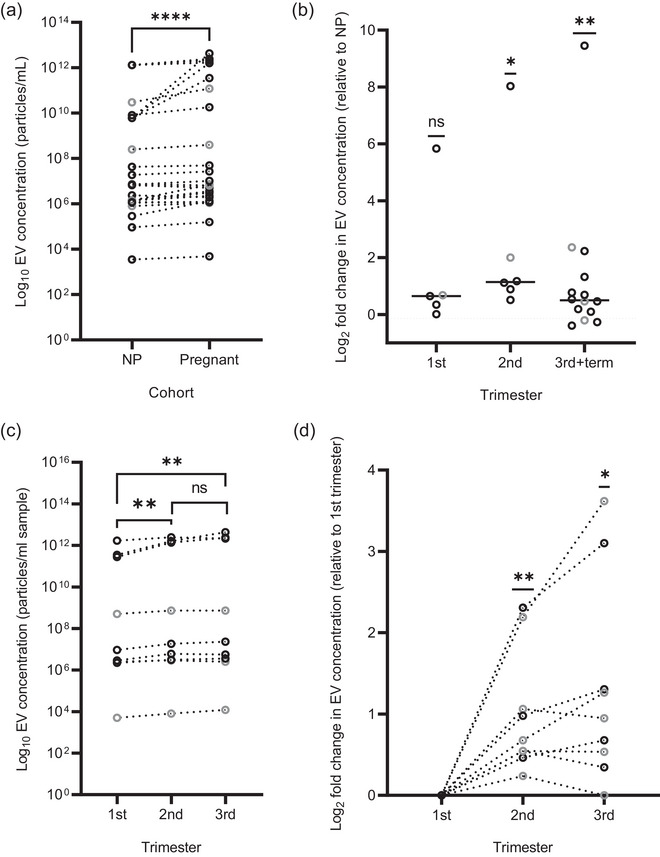
EV concentrations in non‐pregnant controls and healthy pregnancies, and across gestation. (a) Total EV concentrations in the serum/plasma of healthy pregnant and non‐pregnant women compared by Wilcoxon matched‐pairs signed rank test. (b) Log_2_ fold‐change in EV concentrations in pregnant women organised by trimester compared to corresponding non‐pregnant control group and analysed by One‐sample Wilcoxon test. (c) EV concentrations across healthy pregnancy from longitudinal studies. Trimesters compared by Friedman test with Dunn's multiple comparisons. (d) Log_2_ fold‐change in EV concentrations in the second and third trimester compared to the first trimester, analysed by One‐sample Wilcoxon test.* *P* ≤ 0.05, ** *P* ≤ 0.01, **** *P* ≤ 0.0001. Datapoints presented in grey were extrapolated from published graphs. NP: non‐pregnant.

The reported EV concentration in blood ranged between 3.53 × 10^3^–1.33 × 10^12^ particles/mL in non‐pregnant and 4.87 × 10^3^–4.30 × 10^12^ particles/mL in healthy pregnant women. The overall mean and median concentrations in non‐pregnant women were 1.62 × 10^11^ particles/mL and 7.06 × 10^6^ particles/mL, respectively, and in healthy pregnant women were 5.08 × 10^11^ particles/mL and 7.20^6^ particles/mL, respectively. EV concentrations in the blood of healthy pregnant women were 3.14‐fold higher than that of non‐pregnant controls (*P* < 0.0001, *n* = 19, Figure 6a). Separating EV quantification data by pregnancy trimester showed higher levels of EVs in the first trimester (*n* = 5/5, *P* = 0.063), second trimester (*n* = 6/6, *P* = 0.031) and third trimester/term (*n* = 11/14, *P* = 0.009) compared to non‐pregnant controls (Figure 6b). Whilst most studies reported < 5‐fold increase of EVs in pregnancy, one study reported exceptionally high fold‐changes of 57, 261 and 701 in the first, second and third trimesters, compared to non‐pregnant controls, respectively (Salomon et al., 2014). Exclusion of this study from the analysis resulted in the second trimester no longer having statistically significantly higher levels of EVs (*P* = 0.063) compared to non‐pregnant, whilst the difference at third trimester, for which there were more studies, remained significantly higher (*P* = 0.017). Nine studies quantified EV concentrations longitudinally across gestation (Campello et al., 2018; Lok et al., 2009a; Menon et al., 2020; Orozco et al., 2009; Radu et al., 2015, 2017, 2016; Rodrigues et al., 2021; Salomon et al., 2014), enabling us to perform paired longitudinal analysis. A significant increase in EV concentration was observed from the first to the second trimester (*P* = 0.01), and from the first to the third trimester (*P* = 0.007), Figure 6c). EV concentrations between the second and third trimester were not statistically different (*P* > 0.99). Two studies from the same group reported a much higher increase across gestation (Salomon et al., 2014, 2016). These two studies employed Exo‐ELISA as their quantification technique, whereas the others utilised NTA (*n* = 3, Menon et al., 2020; Rodrigues et al., 2021; Salomon et al., 2017) or FC (*n* = 4, Campello et al., 2018; Lok et al., 2008; Orozco et al., 2009; Radu et al., 2015). To account for this inter‐study variation, we therefore also calculated the Log_2_ fold‐increase in EV concentrations in the second and third trimesters compared to the first trimester for each study, which showed a significant increase in EVs between the first and second (*P* = 0.004), and first and third (*P* = 0.013) trimesters (Figure 6d).

### 3.10 Circulating EVs in pregnancy pathologies

Quantification of EVs in women with pregnancy‐ or pre‐existing pathologies compared to healthy controls was performed in 43 studies. One study did not include a healthy pregnant control group (Abolbaghaei et al., 2021). Pathologies included were: pre‐eclampsia (PE; *n* = 27) (Alasztics et al., 2021; Alijotas et al., 2012; Campello et al., 2015; Dragovic et al., 2013; Hu et al., 2018; Jadli et al., 2017; Kovács et al., 2018a, 2018b; Lalic‐Cosic et al., 2021; León et al., 2021; Li et al., 2020; Lok et al., 2007, 2008, 2009b; Maduray et al., 2020; Marques et al., 2012; Mikhailova et al., 2014; Murugesan et al., 2022; Pillay et al., 2016, 2020; Salomon et al., 2017; Textoris et al., 2013; Truong et al., 2017; VanWijk et al., 2002a; Verma et al., 2018; Wang et al., 2020; Weingrill et al., 2021; Zhang et al., 2018), small for gestational age/fetal growth restriction/intrauterine growth restriction (SGA/FGR/IUGR; *n* = 5) (Alijotas et al., 2012; Bretelle et al., 2003; Jadli et al., 2017; Li et al., 2020; Miranda et al., 2018), pre‐term birth or premature pre‐term rupture of membranes (PTB/PPROM; *n* = 4) (Menon et al., 2020; Menon, Debnath et al., 2019; Tronco et al., 2020; Truong et al., 2017), PE with IUGR (*n* = 2) (Alijotas et al., 2012; Jadli et al., 2017), gestational diabetes mellitus (GDM; *n* = 4) (James‐Allan et al., 2020; Jiang et al., 2022; Salomon et al., 2016; Zhang et al., 2021), pregnancy loss/recurrent miscarriage/unexplained fetal loss (PL/RM/UFL; *n* = 2) (Alijotas‐Reig et al., 2011; Monteiro et al., 2020), Type‐1 diabetes (*n* = 1) (Abolbaghaei et al., 2021), HIV with or without PE (*n* = 1) (Pillay et al., 2020), HIV with or without malaria (*n =* 1) (Moro et al., 2016) and anti‐phospholipid syndrome (APS; *n* = 1) (Campello et al., 2018). Of the PE studies, three looked at both early‐ and late‐onset PE (EOPE/LOPE) (Maduray et al., 2020; Pillay et al., 2016, 2020) and one study included cohorts of both mild/moderate and severe PE (Textoris et al., 2013).

### 3.11 Changes in circulating EV concentrations in pregnancy pathologies/pre‐existing morbidities

**FIGURE 7:**
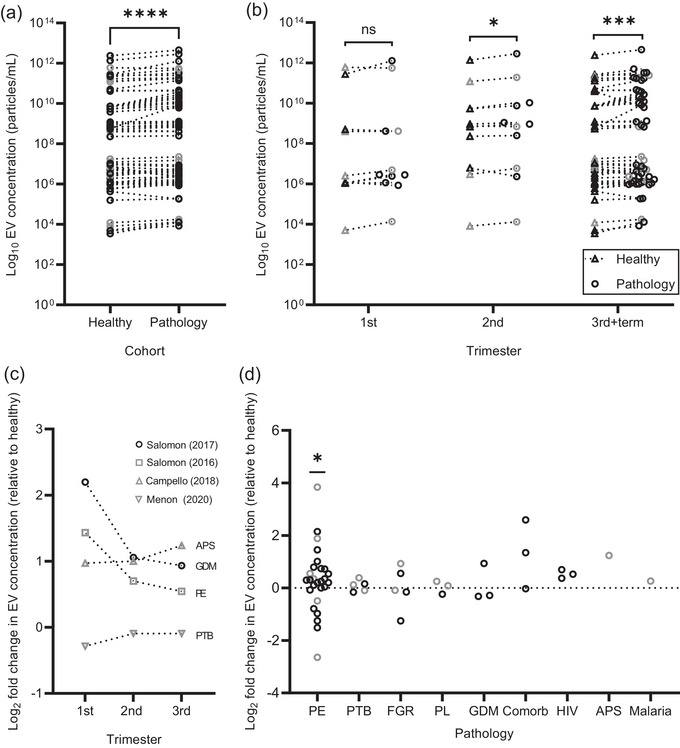
EV concentrations in healthy and pathological pregnancies. (a) EV concentrations of both healthy and pathological pregnancies (*n* = 42), regardless of gestation, plotted on a Log_10_ scale and analysed by Wilcoxon matched‐pairs signed rank test. (b) EV concentrations organised by trimester of pregnancy and compared to the corresponding healthy pregnant control group by Wilcoxon matched‐pairs signed rank tests. (c) The fold change in EV concentrations from pathological pregnancies to healthy pregnant controls plotted for each trimester. (d) Log_2_ fold change in EV concentrations in the third trimester relative to healthy pregnant controls organised by pathology and analysed by One‐sample Wilcoxon test. * = *P* ≤ 0.05, *** = *P* ≤ 0.001, **** = *P* ≤ 0.0001. Grey datapoints represent values extrapolated from published graphs. APS: anti‐phospholipid syndrome, Comorb: co‐morbidities, FGR: fetal growth restriction, GDM: gestational diabetes mellitus, PE: pre‐eclampsia, PL: pregnancy loss, PTB: pre‐term birth.

Comparison of EV concentration in all pathologies compared to their respective healthy pregnant controls found an overall 1.86‐fold higher EV concentration in the pathological group (Figure 7a; *P* < 0.001). Splitting by gestation (Figure 7b) revealed a significant increase in EV concentrations in pathological pregnancies in the second trimester and third trimester/term (*P* = 0.024 and *P* = 0.001, respectively) but not in the first trimester (*P* = 0.365). Four studies looked at EV concentrations in pathological pregnancy cohorts across the three trimesters of pregnancy compared to gestational age‐matched healthy controls (Figure 7; Campello et al., 2018; Menon et al., 2020; Salomon et al., 2016, 2017). In PE (Salomon et al., 2017) and GDM (Salomon et al., 2016), the greatest increase in EV concentrations between pathology and normal pregnancy was seen in the first trimester (2.7‐ and 4.58‐ fold increase, respectively), dropping in the second (1.63‐ and 2.08‐fold increase, respectively) and third trimester (1.46‐ and 1.91‐fold increase, respectively), though remaining higher than the controls. In patients with anti‐phospholipid syndrome (APS) (Campello et al., 2018), circulating EV levels were elevated, relative to healthy controls, as gestation proceeded, whilst patients who delivered preterm (spontaneous delivery < 37 weeks gestation) (Menon et al., 2020) showed a small decrease in the first trimester, but no change for the second and third trimesters. For the most commonly studied third trimester, we separated all relevant studies by pathology, calculating the Log_2_ fold‐change in EV concentration between pathological and healthy pregnancies to account for inter‐study variation in isolation and quantification (Figure 7d). Combined analysis of study‐reported mean EV concentrations found that only concentrations of vesicles measured in PE exhibited a statistically significant increase compared to the healthy controls (*P* = 0.038). For many pathologies, fold‐changes both >1 and <1 were reported (results summarised in Table S2). Despite PE being widely reported to be associated with increased circulating EVs, this is not always the case, with three studies (Lok et al., 2008, Lok et al., 2009b; Pillay et al., 2016) reporting a two‐fold decrease in EV levels in PE compared to healthy controls in the third trimester, and less than a quarter of the studies (18%; 4/22) reporting more than a two‐fold increase (Kovács et al., 2018a, 2018b; Pillay et al., 2016, 2020; Verma et al., 2018).

### 3.12 Cellular source of circulating EVs in pregnancy

**FIGURE 8:**
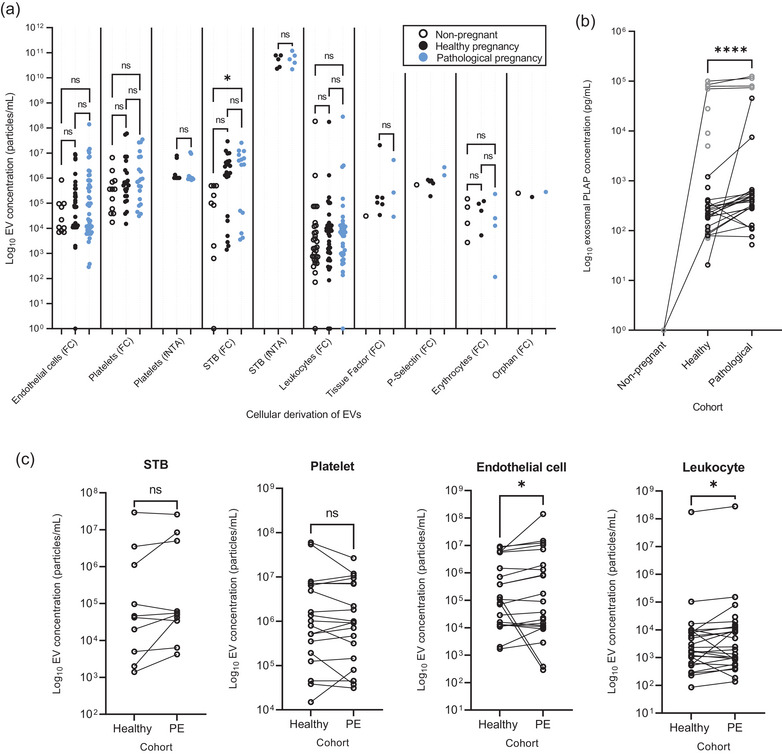
Cell‐specific EVs in non‐pregnant and healthy and pathological pregnancies. (a) EV counts plotted on a Log_10_ scale for each parental cell type identified. The method of quantification indicated in parentheses (*n* = 54). Orphan EVs are those with no identified origin. (b) Quantification of placental‐derived vesicles, by PLAP ELISA, plotted on a Log_10_ scale for healthy and pathological pregnancies, regardless of gestational timepoint. (c) Concentration of EVs derived from the placenta, platelets, endothelial cells and leukocytes (from left to right) plotted on a Log_10_ scale for both healthy pregnancies and those complicated by PE (3rd trimester). * = *P* ≤ 0.5, **** = *P* ≤ 0.0001, Wilcoxon matched‐pairs signed rank test. ELISA: enzyme‐linked immunosorbent assay, FC: flow cytometry, fNTA: fluorescence nanoparticle tracking analysis, PE: pre‐eclampsia, PLAP: placental alkaline phosphatase, STB: syncytiotrophoblast.

Investigation of EV populations deriving from a specific cell type, via surface marker phenotyping, was performed in 58/152 studies. We reviewed the quantification data from 54 of these, as the remaining four presented data as fold‐change, % of EVs of specific cellular derivation, flow cytometric plots without numbers, or without absolute counts of particles (Pap et al., 2008; Tersigni et al., 2021; Walenta et al., 2012; Wong et al., 2015). The concentration of cell‐specific EVs in the blood of healthy non‐pregnant, healthy pregnant, or pathological pregnant participants, measured by either flow cytometry (FC) or fNTA, are summarised in Figure 8a. Cellular sources included endothelial cells (*n* = 22 (FC)) (Abolbaghaei et al., 2021; Alijotas‐Reig et al., 2011, 2012; Bretelle et al., 2003; Buca et al., 2022; Campello et al., 2018; Chen et al., 2012; Ferrari et al., 2022; González‐Quintero et al., 2003, 2004; Hu et al., 2018; Jadli et al., 2019; Lalic‐Cosic et al., 2021; Ling et al., 2014; Lok et al., 2008; Marques et al., 2012; Radu et al., 2015; Salem et al., 2015; VanWijk, Nieuwland et al., 2002a; Zhang et al., 2018), platelets (*n* = 23 (FC), (Abolbaghaei et al., 2021; Alasztics et al., 2021; Alijotas‐Reig et al., 2011, 2012; Bretelle et al., 2003; Buca et al., 2022; Campello et al., 2015, 2018; Dragovic et al., 2013; Ferrari et al., 2022; González‐Quintero et al., 2003; Hu et al., 2018; Kovács et al., 2018a, 2018b; Lalic‐Cosic et al., 2021; Lok et al., 2008; Marques et al., 2012; Radu et al., 2015; Salem et al., 2015; Textoris et al., 2013; VanWijk et al., 2002b; Zhang et al., 2018; Chen et al., 2021b), the syncytiotrophoblast (*n* = 11 (FC) (Dragovic et al., 2013; Ferrari et al., 2022; Gill et al., 2019; Hu et al., 2018; Kovács et al., 2018a, 2018b; Lok et al., 2008; Marques et al., 2012; Moro et al., 2016; Orozco et al., 2009; VanWijk et al., 2002b; Chen et al., 2021b), *n* = 3 (NTA) (James‐Allan et al., 2020; Miranda et al., 2018; Menon, Dixon et al., 2019), *n* = 14 (ELISA)) (Dragovic et al., 2013; Goswami et al., 2006; Germain et al., 2007; Knight et al., 1998; Maduray et al., 2020; Murugesan et al., 2022; Pillay et al., 2016, 2020; Reddy et al., 2008; Salomon et al., 2016, 2017; Sarker et al., 2014; Salomon et al., 2014; Verma et al., 2018), leukocytes (*n* = 14 (FC)) (Abolbaghaei et al., 2021; Alijotas‐Reig et al., 2011, 2012; Buca et al., 2022; Campello et al., 2015; Lok et al., 2009a; Dragovic et al., 2013; Ferrari et al., 2022; Marques et al., 2012; Mikhailova et al., 2014; Radu et al., 2015; VanWijk et al., 2002a; VanWijk et al., 2002b; Zhang et al., 2018), tissue factor‐bearing (*n* = 4 FC)) (Alasztics et al., 2021; Campello et al., 2015; Lalic‐Cosic et al., 2021; Radu et al., 2015), P‐Selectin‐bearing (*n* = 3 FC)) (Campello et al., 2015; Radu et al., 2015; Alasztics et al., 2021), erythrocytes (*n* = 4 (FC)) (Dragovic et al., 2013; Hu et al., 2018; Marques et al., 2012; VanWijk et al., 2002b) and ‘orphan’ (*n* = 1, FC)) (Dragovic et al., 2013). There were also studies that included other markers such as VCAM‐1 and PIGF (Lalic‐Cosic et al., 2021), or EpCAM (Buca et al., 2022).

Fourteen studies utilised ELISA to measure placental‐derived EVs through PLAP detection (Figure 8b), with five of these studies including multiple pathologies and timepoints (Germain et al., 2007; Pillay et al., 2016, 2020; Salomon et al., 2014, 2017). EV‐associated PLAP was undetectable in the blood of non‐pregnant women by ELISA, and a significant (*P* < 0.0001) increase in PLAP concentration was measured in pathological pregnancy cohorts compared to healthy pregnant controls (Figure 8b). All of these studies used the marker PLAP; five utilising an in‐house monoclonal antibody NDOG2 (Dragovic et al., 2013; Goswami et al., 2006; Germain et al., 2007; Knight et al., 1998; Reddy et al., 2008), generated by (Davies et al., 1985), and the remainder employing commercial PLAP ELISA kits (Maduray et al., 2020; Murugesan et al., 2022; Pillay et al., 2016, 2020; Salomon et al., 2014, 2016, 2017; Sarker et al., 2014; Verma et al., 2018).

In contrast, when flow cytometry was employed, PLAP‐positive EVs were reported in the non‐pregnant controls of five out of the six studies (Chen et al., 2021a; Dragovic et al., 2013; Marques et al., 2012; Orozco et al., 2009; VanWijk, Nieuwland et al., 2002a), which could be due to antibodies binding to non‐placental alkaline phosphatases, or other non‐specific binding/autofluorescence. Due to small sample sizes, we could only compare the cellular source of EVs derived from placental tissue, platelets, endothelial cells, or leukocytes between healthy and PE cohorts in the third trimester for statistical analysis. Overall, endothelial cell‐ and leukocyte‐derived EVs were significantly increased in PE (*P* = 0.025 and 0.013, respectively, Figure 8c).

### 3.13 EV size distribution in non‐pregnant, pathological, and healthy pregnant cohorts

**FIGURE 9:**
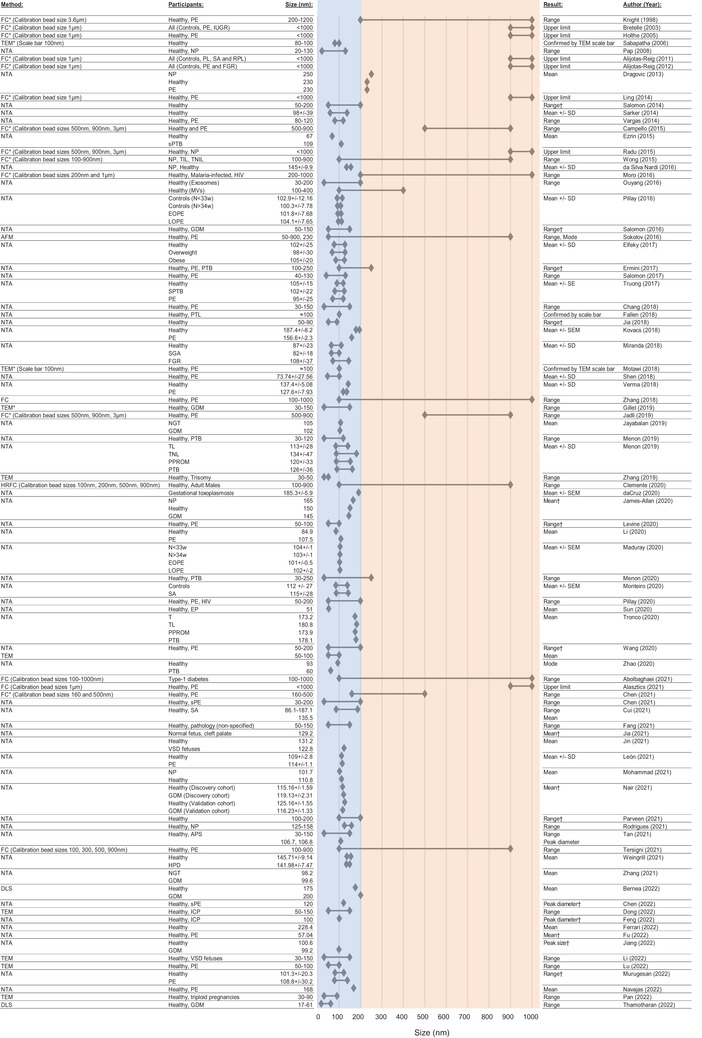
Size distribution of circulating EVs from pregnant and non‐pregnant women. The orange and blue bars represent the size distributions of small (<200 nm) and medium/large EVs (>200 nm), respectively. The black arrows show the sizes of the vesicle preparations from each study. ‘†’ indicates the size was also confirmed by TEM, as a validatory method. AFM: atomic force microscopy, DLS: dynamic light scattering, EM: electron microscopy, EP: ectopic pregnancy, FC: flow cytometry, FGR: fetal growth restriction, GDM: gestational diabetes mellitus, HRFC: high resolution flow cytometry, NGT: normal glucose tolerance, NP: non‐pregnant, NTA: nanoparticle tracking analysis, PE: pre‐eclampsia, PROM: premature rupture of membrane, PTB: pre‐term birth, RM: recurrent miscarriage, SA: spontaneous abortion, SD: standard deviation, SGA: small for gestational age, TIL: term in labour. TNIL: term not in labour, UFL: unexplained fetal loss.

The size distribution of circulating EVs was reported in 83 studies (Figure 9). For the 45 studies that referred to their EV preparations as ‘exosomes’, all presented the average sizes as primarily ranging from 30–200 nm, encompassing the size range of not only exosomes, but also other EVs, including microvesicles. 16/19 studies that employed the term ‘EV’ reported EVs sizing between 30–250 nm (Dragovic et al., 2013; da Cruz et al., 2020; Ezrin et al., 2015; Fallen et al., 2018; Ferrari et al., 2022; Gillet et al., 2019; James‐Allan et al., 2020; Kovács et al., 2018a, 2018b; Levine et al., 2020; Murugesan et al., 2022; Nair et al., 2021; Nardi et al., 2016; Pan et al., 2022; Thamotharan et al., 2022; Tronco et al., 2020; Zhang et al., 2019) as measured by NTA (*n* = 12), TEM (*n* = 3) and DLS (*n* = 1). The other three studies that used the term ‘EV’ used FC to quantify and determined that their vesicles were between either 100–500 nm or 100–900 nm in size (Alasztics et al., 2021; Chen et al., 2021a; Clemente et al., 2020). The larger reported size ranges could be due to calibration bead sizes and the limited resolution of standard cytometers. For example, one study (Clemente et al., 2020) used nanoscale high resolution flow cytometry with a lower limit of detection at 100 nm, and another (Chen et al., 2021a) included calibration beads of 160, 200 and 240 nm. Three studies utilised the term ‘sEV’ to describe their EVs and reported sizes of 100–150 nm (León et al., 2021; Mohammad et al., 2021; Rodrigues et al., 2021).

The majority of the studies reported no difference in the size of EVs obtained from the blood of women with pregnancy complications (PE, PTB, FGR, GDM, obesity) compared to healthy pregnancies (Dragovic et al., 2013; Elfeky et al., 2017; James‐Allan et al., 2020; Kovács et al., 2018a, 2018b; Menon et al., 2020; Miranda et al., 2018; Pillay et al., 2016). Only one study reported significantly larger EVs in their PE cohort (107.5 nm) compared to their healthy pregnant controls (84.9 nm) (Li et al., 2020). These analyses are complicated by the presence of contaminating lipoproteins. Overall, the results collated from the included studies were not conclusive as to whether the size of EVs could be a contributing biomarker for pregnancy‐related pathologies.

## 4 DISCUSSION

Extracellular vesicles (EVs) have garnered increasing attention as a potential source of novel biomarkers for pregnancy pathologies, and maternal blood is an especially attractive source for EV isolation. However, inter‐study differences in EV isolation and characterization methodologies could hamper the applicability of identified biomarkers across different study populations. In this comprehensive review, we identified 152 studies published between 1998 and 2022 that have investigated EVs in the circulation of pregnant women. We have meticulously explored the heterogeneity of methodology, technical implications of varying protocols, and performed combined analysis of EV concentrations across gestation and in pathological pregnancies. Our findings reveal that there is high variability between studies in terms of pre‐processing of blood samples, EV isolation methods, and EV characterisation techniques. This variability is reflected in a wide range of reported EV concentrations in pregnant blood. Despite this variability, we identified an overall significant increase in EV populations in pregnancy compared to non‐pregnant age‐matched controls. Our analysis also suggests that EV concentrations were significantly higher with increased gestational age and in pathological pregnancies. However, these data only highlight the possibility that EV concentration could be used as a predictive biomarker for pregnancy pathologies. Given the high variability in reported EV/particle concentrations, and the issue of lipoprotein contamination, we believe that the field is currently far from being able to recommend a threshold of ‘normal’ EV concentration in pregnancy. We believe that specific EV cargo are more likely to be useful as clinical biomarkers. We have further reviewed the literature reporting cell‐specific EVs based on surface markers, including placental EVs, and have noted that placental‐derived EVs were often, but not always, increased in pathological pregnancies. This review emphasizes the need for detailed reporting of EV isolation and characterization methods in future EV studies in pregnancy. Furthermore, there is a pressing need for standardization to enable the identification of robust biomarkers for pregnant populations. Our findings provide an important framework for future studies aimed at identifying novel biomarkers for pregnancy pathologies.

Our heterogeneity plot visualises the methodology of 152 studies, and shows wide variation, and temporal shifts, in favoured nomenclature, anticoagulant, EV isolation and characterisation methods. Heterogeneity has also been shown in other studies that compared the pre‐analytical conditions of EV preparation in multiple types of body fluids, including blood, urine, breast milk, etc, highlighting the need for standardisation across all EV work (López‐Guerrero et al., 2023). Most studies in our review utilised plasma as their starting material, with EDTA and sodium citrate being the most commonly used anticoagulants. Plasma is generally preferred for EV isolation due to the potential release of additional EVs during serum clotting, (Wolf, 1967; Coumans et al., 2017), and indeed, the highest EV concentration reported in maternal blood (8.7 × 10^12^/mL) was from a study utilising serum (Jia et al., 2018). There are currently no pregnancy‐specific studies that have investigated the effect of pre‐processing methodology on blood EV parameters. However, investigations into the effects of pre‐processing parameters on the concentration of EVs measured in biological samples showed that, following the collection of blood, delay in first centrifugation by as little as one hour increased the detection of Annexin‐V+ EVs (Lacroix et al., 2012). Furthermore, freezing of platelet‐containing blood samples cause the release of *ex vivo* EVs (Kim et al., 2022), and the freezing of plasma samples can also decrease the number of detectable EVs compared to fresh samples (Gelibter et al., 2022), highlighting the importance of recording every stage of sample processing and the potential implications these parameters have in detecting physiological blood EVs. In the 123 studies included in our analyses that utilised plasma, only 37% (*n* = 45) recorded the time between blood collection/plasma preparation and EV isolation, whilst 76% (*n* = 93) recorded whether the plasma samples were frozen before EV isolation.

*Ex vivo* release of platelet EVs in plasma samples is a well known issue in the field of blood EVs, and is exacerbated through platelet activation. Through our analysis, we found that EDTA plasma samples were reported to have higher and more variable levels of EVs compared to citrated plasma. However, other studies report that EDTA stabilises the concentration of platelet EVs following blood collection from non‐pregnant patients compared to citrate (Buntsma et al., 2021), suggesting that this increase could be of non‐platelet EVs, or could be due to other coincident variabilities in blood processing between laboratories. Outside of pregnancy, a previous systematic analysis (EV Blood Benchmarking (EVBB) study) of various blood collection tubes and blood processing intervals highlighted the implications that these have on platelet activation (and subsequent increases in platelet EVs), overall EV concentration, and EV contents (protein and RNA) (Dhondt et al., 2023). This study concluded that citrate and ACD‐A blood tubes performed the best across all parameters. Previous studies reported that citrate tubes caused less platelet activation (Mussbacher et al., 2017), as platelet activation can stimulate the release of platelet‐derived EVs (Wei et al., 2018), and blood collected into ACD‐A tubes yielded less platelet‐derived EVs in the platelet‐depleted plasma than EDTA or heparin tubes (György et al., 2014). Furthermore, it's not just platelets and platelet‐derived EVs that seem to be impacted by blood collection tube, as it has also been shown that blood processed by heparin or EDTA resulted in the greatest increase in red blood cell‐derived EVs, compared to citrated tubes (Makhro et al., 2016). It has been suggested that a minimum of two centrifugation steps is required for platelet removal from plasma (Coumans et al., 2017), but only a small proportion of pregnancy studies report performing this. We conclude that a direct comparison study of different anticoagulants, and blood processing protocols, is needed for maternal blood samples, guided by the work performed in the EVBB study (Dhondt et al., 2023). As stressed by many researchers in the EV field, pre‐quantification parameters clearly need to be considered when determining the clinical importance of circulating EV concentrations from multiple studies.

EV nomenclature is an important issue, as highlighted by the MISEV guidelines, and their update (Lötvall et al., 2014; Théry et al., 2018). Specific to the pregnancy field is the historical utilisation of the term STBM, denoting syncytiotrophoblast microvesicles or microparticles. Of note, some studies utilise this term regardless of whether they have shown syncytiotrophoblast derivation of EVs. The term STBM has become less popular in recent years, which have seen the increased use of the term ‘exosome’ and ‘extracellular vesicle’. Of note, the vast majority of studies utilising the term ‘exosome’ have not employed isolation methods that would specifically isolate exosomes. The MISEV 2018 update (Théry et al., 2018) recommends the use of the term ‘extracellular vesicle (EV)’ for vesicle preparations, and sub‐categorising them based on their size, biochemical composition, or description of cellular origin, as needed. This appears to have been adopted by the field, with the term ‘extracellular vesicle’ becoming more popular in recent years, particularly since 2019, as well as four studies published in 2021/2022 using the term small EVs (sEV), and one study from 2021 using the term large EV (lEV), This is an area that requires improvement in the pregnancy field. There was a high degree of variability and overlap between isolation methods and nomenclature , highlighting the importance for readers to take note of EV isolation methodology when interpreting findings of pregnancy studies.

EV‐TRACK is one platform which assists with reporting of EV methodology, and is aimed at improving reproducibility and aiding interpretation of EV research (Van Deun et al., 2017). By submitting 123 studies to EV‐TRACK, we scored a total of 128 eligible studies against their criteria, identifying specific areas of methodological reporting requiring improvement. We found that the measurement of EV‐enriched proteins was the most frequently reported experimental parameter, with 61% of studies meeting this criterion. However, other important parameters, such as EV density, lysate preparation, and electron microscopy images, were reported less frequently, with scores of 30% or lower. Future studies of circulating EVs in pregnancy should therefore endeavour to include reporting of these parameters to improve reproducibility in the field, particularly given the high variability in the methods utilised that we report, impeding the direct comparison between study populations. As, prior to our efforts, 96.1% of pregnancy studies were not present on EV‐TRACK, we also encourage use of this platform to improve future reporting and reproducibility.

We found that differential ultracentrifugation (DUC) has historically been a popular method of EV isolation from pregnant blood, but that the proportion of studies utilising DUC as a single isolation method has decreased over time, with a corresponding increase in the use of various other isolation methods. Currently, there is no apparent preferred technique in this field, with SEC, membrane filtration, and immuno‐capture all being utilised in recent years, among others. The purity and characteristics of EVs isolated by various methods have been previously reported (Witwer et al., 2013; Andreu et al., 2016; Kırbaş et al., 2019; Zhang et al., 2020). PEG‐based precipitation (including commercial reagents ExoQuick™ and Total Exosome Reagent™), which co‐isolates non‐EV contaminants, such as lipoproteins (Ludwig et al., 2018; Brennan et al., 2020; Pavani et al., 2020), was used by a large proportion of pregnancy studies (38%; *n* = 57). More problematically, these PEG‐isolated EVs were commonly called exosomes, which should be interpretated with caution.

It has been suggested that combining isolation methods could enhance purity (Théry et al., 2018). As an example of this, three pregnancy studies that utilised PEG also employed an additional subsequent DUC step of 100,000*xg* to potentially help precipitate EVs from non‐EV contaminants (Ermini et al., 2017; Ouyang et al., 2016; Shen et al., 2018). Several studies have also incorporated additional isolation techniques such as sucrose cushions or iodixanol gradients to separate vesicles according to density, particularly in recent years. The choice of isolation method may impact downstream analyses and interpretation of results; thus, pregnancy researchers should carefully consider their choice of EV isolation method, taking into account the potential advantages and disadvantages of each approach.

The reported concentration of EVs varied widely, with concentrations of 4.87 × 10^3^–4.30 × 10^12^ particles/mL in pregnant blood compared to 3.53 × 10^3^–1.33 × 10^12^ particles/mL in non‐pregnant blood. These differences could be due to many methodological differences between study sites in terms of patient characteristics, sample collection, sample pre‐processing, EV isolation and EV quantification. In differential centrifugation‐isolated EVs, flow cytometry (FC) yielded significantly lower EV counts compared to nanoparticle tracking analysis (NTA). While FC is more accurate in terms of using EV‐specific surface markers, its size detection limit is restricted by the size of calibration beads (Welsh et al., 2020), and the resolution of the flow cytometer makes it difficult to detect smaller vesicles. Conversely, nanoparticle tracking analysis (NTA) may overestimate EV counts by including non‐EV particles such as lipid/protein aggregates (Desgeorges et al., 2020). For instance, Dragovic et al., 2013 reported 100‐fold greater EV numbers using NTA (4 × 10^8^) compared to FC (1.2 × 10^6^). We, therefore, need to consider that lipoproteins are often being quantified alongside EVs, particularly when the quantification techniques do not distinguish EVs from other extracellular particles. For example, Annexin‐V and lactadherin can label both EVs and lipoproteins during flow cytometry (Botha et al., 2022), but the combination of both Annexin‐V with light scatter can be used to distinguish EVs from lipoproteins.

Based on the quantification results in the current literature, it is hard to determine the true normal and abnormal concentrations of EVs in healthy and pathological pregnancies. To address these limitations, recent developments in quantification techniques have combined the strengths of both FC and NTA. Fluorescence nanoparticle tracking analysis (fNTA) uses quantum dots (Qdots) to detect EVs labelled with fluorescent antibodies, allowing quantification and profiling while also measuring sample purity (Thane et al., 2019). This technology can also determine the percentage of detected particles that are positive for a specific marker, allowing for assessment of sample purity. For example, Truong et al. reported that 92 ± 5% of their isolated EVs were positive for CD63 (Truong et al., 2017). This could also help with determining physiological changes in EVs across pregnancy‐related pathologies, as it could be used to measure changes to the size of labelled EVs, as opposed to potentially contaminating lipoproteins in unlabelled EV isolations. However, fNTA has limitations, such as issues with optimization (Thane et al., 2019), and the inability to label more than one surface marker at a time, which precludes analysis of co‐expression of surface markers. Another newer technology is specialised nanoscale flow cytometers that can measure smaller EVs than conventional flow cytometers, and can differentiate background ‘noise’ from 100 nm calibration beads, making them suitable for detecting sEVs (Morales‐Kastresana et al., 2017). This is likely to be a future area of interest for the pregnancy field.

ELISAs are another commonly used method for characterising EVs by targeting specific surface markers. ExoELISA™ is a commercially available assay that quantifies vesicles based on the presence of CD63 and uses a standard curve to determine vesicle concentration. Although this technique allows measurement of CD63+ EVs, it supposes a standardised number of CD63 molecules per EV, and the heterogeneity of EV surface markers likely means it is not particularly suitable for total EV concentration. Despite this limitation, two studies reported that the concentration of EVs measured by NTA and ExoELISA were consistent (Maduray et al., 2020; Pillay et al., 2016). ELISAs can also be utilised to measure cell‐specific EV populations, and 14 pregnancy studies utilised them to measure placental‐derived EVs, using PLAP as the protein marker. Of note, PLAP and other placental markers, including HLA‐G and syncytin‐1, are also upregulated in malignancy (Lin & Yan, 2018; Liu et al., 2019; Reiswich et al., 2021), and PLAP antibodies can cross‐react with other alkaline phosphatases, as exemplified by PLAP+ EVs being reported in the blood of non‐pregnant individuals. It is thus very important to include non‐pregnant controls in all studies to check the specificity of any antibodies being utilised to measure or isolate placental EVs.

In confirmation of reports from several individual studies, our meta‐analysis found that EV concentrations are elevated in pregnancy and increase as pregnancy proceeds. Across all 19 studies that compared pregnant and non‐pregnant blood, the concentration of EVs in the blood of healthy pregnant women was three times higher, but results were variable. Three studies showed no change or even a decrease in EV concentration (Lok et al., 2007, 2008; VanWijk, Nieuwland et al., 2002a), but one third of studies (6/19) reported a fold‐increase of > 2 in pregnancy compared to non‐pregnant controls (James‐Allan et al., 2020; Mikhailova et al., 2014; Mohammad et al., 2021; Pap et al., 2008; Radu et al., 2015; Salomon et al., 2014). These inter‐study variations could reflect methodological differences that result in preferential isolation of certain EV subsets that are enriched/depleted in pregnancy. When pregnant data were spilt out by gestation, significantly higher EV levels were detected in the second and third trimesters. The increase in the first trimester did not reach statistical significance, potentially due to small study numbers at this gestation (*n* = 5). Both of our meta‐analyses utilising cross‐sectional data, and longitudinal data (*n* = 9) reinforced previous individual reports that EV concentrations increase as pregnancy proceeds. This could reflect both the increased release of EVs from the placenta, which grows rapidly in size throughout pregnancy, or the increased release of EVs from other cells such as activated platelets (Lok et al., 2012; Salomon et al., 2014). The reported change from the first trimester to third trimester varied widely between studies, from no change, up to a 12‐fold increase. There are not many longitudinal studies of circulating EVs across all three trimesters (*n* = 9), a study design that could yield highly useful information, and there is also little granularity beyond trimester grouping.

Another key question has been whether EV concentrations are altered in complications of pregnancy. EVs have been mainly studied in pre‐eclampsia (PE; *n* = 27), with three of these additionally investigating early‐ versus late‐onset PE (Maduray et al., 2020; Pillay et al., 2016, 2020; ) and one looking at mild/moderate versus severe PE (Textoris et al., 2013). There are a few studies investigating other pathologies including growth restriction, pre‐term birth or premature pre‐term rupture of membranes, gestational diabetes, pregnancy loss, and others. Our analysis of the 42 pathological studies with a healthy comparator showed a significant overall increase in total EV concentrations in pathological pregnancies compared to healthy pregnancies, but reported changes again varied widely. Cross‐sectional analysis suggests that differences are more common in the third trimester, but analysis of the few longitudinal studies painted a different picture, depending on the pathology. For example, GDM (Salomon et al., 2016) and PE (Salomon et al., 2017) were shown to have the highest increase in the first trimester, compared to non‐pathological pregnancy. The diagnostic potential of EV concentrations in pregnancy‐related pathologies is difficult to evaluate with the available data, especially given that each study has its own control population, methodology and gestational timepoint. Given the variability, EV concentration alone is likely not going to be a useful tool its utility for early prediction of pregnancy pathologies, though it could aid stratification or be used in combination with other biomarkers.

Pre‐eclampsia (PE) is the most studied pathology in the field, and our combined analysis of the 22 studies that compared EV concentrations to normotensive pregnancies, confirmed that PE is usually associated with higher EV levels. However, less than a quarter of the studies (18%; 4/22) reported more than two‐fold increase (Kovács et al., 2018a, 2018b; Pillay et al., 2016, 2020; Verma et al., 2018). Additionally, three studies reported a two‐fold *decrease* in EV levels in PE compared to healthy controls in the third trimester (Lok et al., 2008; Lok, Christine et al., 2009; Pillay et al., 2016). This again highlights the high variability between studies and the difficulty of translating findings between different study sites.

Fifty‐eight studies quantified cell‐specific EV populations, with 54 of these presenting quantitative data that we collated for further analysis. Endothelial‐ and leukocyte‐derived EVs were significantly higher in PE compared to healthy controls, suggesting EV‐mediated changes to the vasculature and immune function that contributes to the development of PE. The cellular derivatives of EV populations are therefore important to consider mechanistically. Once again, concentrations may be affected by quantification technique, as results for placental, or syncytiotrophoblast‐derived EVs measured by fNTA were significantly higher compared to FC. A previous study analysing technical methodology of EV collection and processing, revealed that very minor changes to a protocol can significantly change the EV concentration obtained from the same blood sample (Ayers et al., 2011); increasing the time between blood collection and initial centrifugation for EV pelleting, increased the amounts of Annexin‐V (+) EVs and platelet‐derived EVs, with the values then plateauing after an hour (Ayers et al., 2011). Only 53/152 studies in this review provided details on the blood sample storage time before EV isolation and/or quantification, and this ranged from immediate processing to storage for up to 48 h.

To conclude, studies of circulating EVs in pregnancy vary widely in terminology, methodology, and conclusions. Additional longitudinal studies could add granularity to our understanding of EV dynamics during pregnancy, and how they change during pathology. Employment of additional placental markers, other than PLAP, could also yield novel data. The two major issues we identified in the field were insufficient reporting of methodology—hindering interpretation and reproducibility—and a mismatch in methodology and terminology. Although EVs provide a potential exciting biomarker for pregnancy‐related pathologies, as presented by the 152 studies included in this review, these issues require addressing in future research. Standardisation of methodology could help resolve these issues but would require a consensus to be reached across different pregnancy groups, and it would require consideration of financial and logistical restraints at different study sites. As a first step, improved methodological rigour and reporting, with help from sources such as MISEV, and EV‐TRACK, will allow us to determine the potential source of inter‐study differences in EV concentrations, and greatly enhance our ability to identify useful biomarkers for pregnancy pathologies.

## AUTHOR CONTRIBUTIONS

**Megan V. C. Barnes**: Data curation; formal analysis; methodology; writing—original draft; writing—review & editing. **Paschalia Pantazi**: Data curation; formal analysis; methodology; supervision; writing—review & editing. **Beth Holder**: Data curation; formal analysis; funding acquisition; methodology; supervision; writing—review & editing.

## CONFLICT OF INTEREST STATEMENT

The authors declare no conflicts of interest.

## Supporting information

Supporting Information

## Data Availability

Data sharing is not applicable to this article as no new data were created or analyzed in this study.
