# Supplementary material for: Circulating extracellular vesicles in healthy and pathological pregnancies: A scoping review of methodology, rigour and results
Source: J Extracell Vesicles. 2023 Nov 16;12(11):12377. doi: 10.1002/jev2.12377 (PMC10654380; doi:10.1002/jev2.12377)
Supplement: Supplementary file 1 — Supporting Information [file JEV2-12-12377-s001.docx]

Figure S1


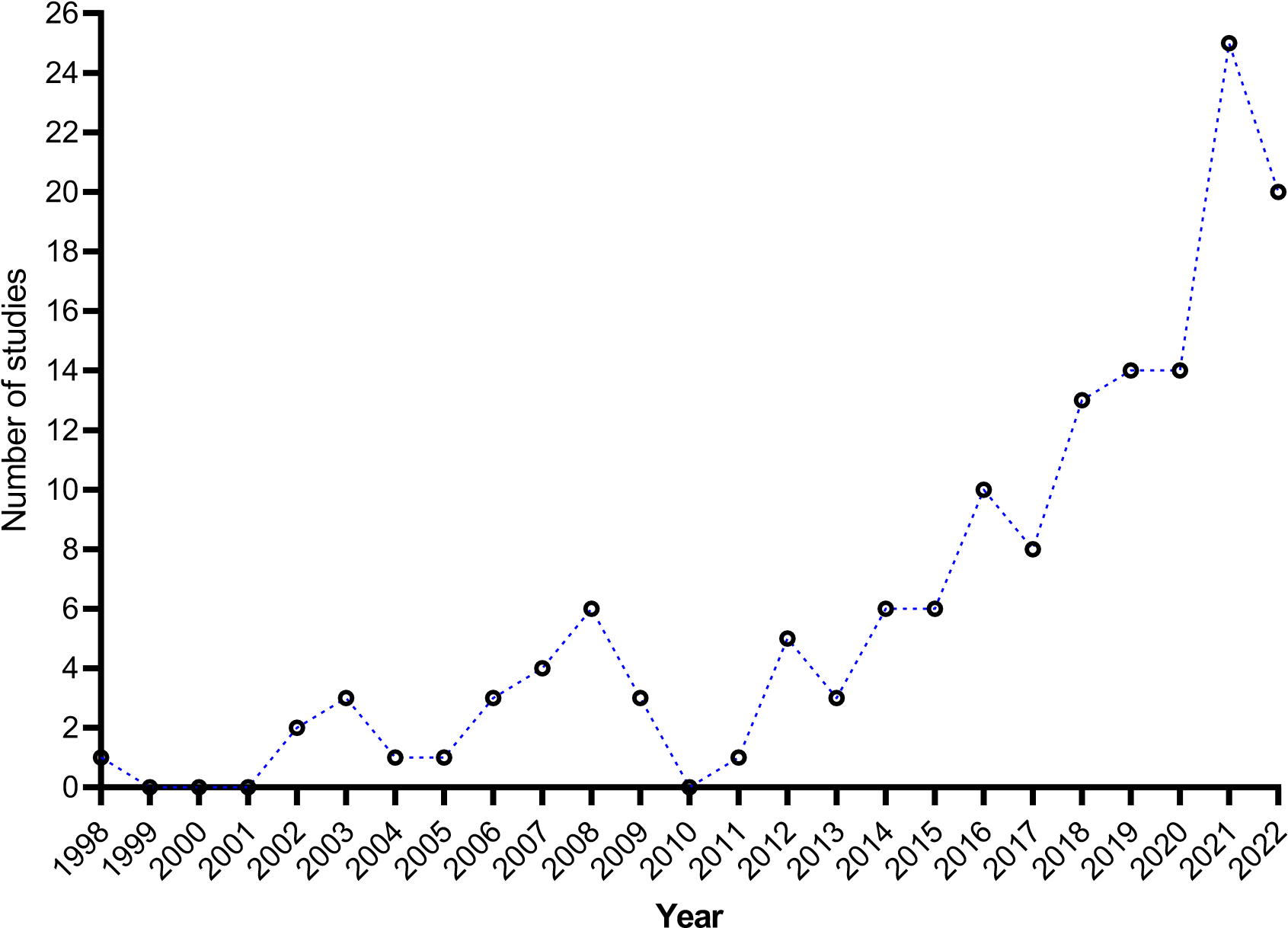


Table S1: Number of studies out of total (152) investigating either healthy, or pregnancy-related pathologies.

| **Cohort:** | | **Number of studies:** | **Additional information:** |
| --- | --- | --- | --- |
| **Healthy pregnancies** | | 152 |  |
| **Pathological pregnancies** | Pre-eclampsia (PE) | 76 | 4 studies measured early onset vs late onset; 3 studies measured mild vs severe PE; 3 studies measured just severe PE. |
|  | Gestational diabetes mellitus (GDM) | 13 |  |
|  | Pre-term birth (PTB)/pre-term premature rupture of the membranes (PPROM) | 12 | 3 studies specifically measured PPROM, either on its own or as a separate cohort to PTB |
|  | Small for gestational age (SGA)/fetal growth restriction (FGR)/intrauterine growth restriction (IUGR) | 11 |  |
|  | Gestational hypertension (GH) | 8 |  |
|  | Pregnancy loss (PL)/Recurrent miscarriage (RM)/Spontaneous  abortion (SA) | 6 |  |
|  | IUGR&PE (Co-morbidity) | 3 |  |
|  | Fetuses with physical abnormalities | 3 |  |
|  | Anti-phospholipid syndrome (APS) | 2 |  |
|  | Overweight (OW)/Obese | 2 |  |
|  | Intrahepatic cholestasis (ICP) | 2 |  |
|  | Fetuses with aneuploidies | 2 |  |
|  | Gestational vascular problems | 1 |  |
|  | Human immunodeficiency virus (HIV) | 1 |  |
|  | HIV&PE | 1 |  |
|  | Placental malaria | 1 |  |
|  | Large for gestational age (LGA) | 1 |  |
|  | Heavy alcohol (EtOH) use | 1 |  |
|  | Toxoplasmosis | 1 |  |
|  | Phthalate exposure | 1 |  |
|  | SARS-CoV-2 convalescence | 1 |  |
|  | Multiple pathologies | 1 | Samples were collected prospectively, and maternal outcomes were followed. Outcomes included: pre-eclampsia, hypertension, proteinuria, and hypoglycaemia. |

Table S2: Summary of EV-quantification results across the studies investigating pregnancy-related pathologies.

**Trimester**

**Pathology**

**Results (total EV concentrations in plasma/serum)**

**Units**

**Method**

**Study**

**Healthy Pathology**

| **PE** | 1st | **4.00E+08** | **4.10E+08** | /mL plasma | NTA | Dragovic (2013) |
| --- | --- | --- | --- | --- | --- | --- |
|  |  | 1.20E+06 | 8.40E+06 | /mL plasma | FC | Dragovic (2013) |
|  |  | 2.90E+09 | N/A | /L plasma † | FC | Lok (2008) |
|  |  | 1.09E+03 | 2.43E+03 | /µL plasma † | FC | Jadli (2017) |
|  |  | **5.00E+03** | **1.35E+04** | /mL plasma | NTA | Salomon (2017) |
|  | 2nd | 5.8E+09 (20 weeks)  5.1E+09 (24 weeks)  7.3E+09 (28 weeks) | 2.3E+09 (28 weeks) | /L plasma † | FC | Lok (2008) |
|  |  | **8.00E+03** | **1.30E+04** | /mL plasma | NTA | Salomon (2017) |
|  |  | 5.40E+09 | 1.06E+10 | /mL plasma | NTA | Truong (2017) |
|  |  | **7.20E+06** | **5.10E+06** | /mL plasma | FC | Mikhailova (2014) |
|  |  | 2.30E+08 | 2.50E+08 | /mL plasma | NTA | Wang (2020) |
|  | 3rd | **1.10E+03** | **1.40E+03** | /µL plasma † | FC | Hu (2018) |
|  |  | 1.10E+03 | 1.10E+03 | /µL plasma † | FC | Alijitos-Reig (2012) |
|  |  | 3.44E+03 | 5.73E+03 | /µL plasma † | FC | Campello (2015) |
|  |  | 3.46E+03 | 1.28E+04 | /mL plasma | FC | Kovacs (2018) |
|  |  | 2.23E+11 | 3.24E+11 | /mL plasma | NTA | Li (2020) |
|  |  | 5.10E+09 | 2.60E+09 | /L plasma † | FC | Lok (2007) |
|  |  | 5.70E+09 (32 weeks)  5.50E+09 (36 weeks) | 3.30E+09 (32 weeks)  2.30E+09 (36 weeks) | /L plasma † | FC | Lok (2008) |
|  |  | **6.50E+09** | **2.30E+09** | /L plasma † | FC | Lok (2008) (2) |
|  |  | **1.20E+04** | **1.75E+04** | /mL plasma | NTA | Salomon (2017) |
|  |  | 1.96E+03 | 2.26E+03 | /L plasma † | FC | VanWijk (2002) |
|  |  | 5.06E+10 | 1.38E+11 | /mL plasma | NTA | Verma (2018) |
|  |  | 1.59E+08 | 1.89E+08 | /L plasma † | FC | Zhang (2018) |
| **EOPE** | 2nd | 9.24E+08 (<33 weeks) | **1.10E+09** | /mL plasma | NTA | Maduray (2020) |
|  |  | 6.84E+08 (<33 weeks) | 9.19E+08 | /mL plasma | ELISA | Maduray (2020) |
|  | 3rd | 5.75E+08 (<33 weeks) | 8.23E+09 | /mL plasma | NTA | Pillay (2016) |
|  |  | 6.16E+09 (<33 weeks) | 2.73E+10 (<33 weeks) | /mL plasma | NTA | Pillay (2020) |
| **LOPE** | 3rd | **1.20 E+09 (>34 weeks)** | 1.30E+09 | /mL plasma | NTA | Maduray (2020) |
|  |  | **1.15E+09 (>34 weeks)** | 1.19E+09 | /mL plasma | ELISA | Maduray (2020) |
|  |  | 3.88E+10 (>34 weeks) | 6.14E+09 | /mL plasma | NTA | Pillay (2016) |
|  |  | 8.45E+09 (>34 weeks) | 1.71E+10 (>34 weeks) | /mL plasma | NTA | Pillay (2020) |
| **Mild PE** | 3rd | 5.95E+02 | 9.79E+02 | /µL plasma † | FC | Textoris (2013) |
| **Severe PE** | 3rd | 5.95E+02 | 1.32E+03 | /µL plasma † | FC | Textoris (2013) |
|  |  | 4.87E+00 | 8.43E+00 | /µL plasma † | FC | Marques (2012) |
| **FGR** | 3rd | 1.10E+03 | 9.96E+02 | /µL plasma † | FC | Alijitos-Reig (2012) |
|  |  | **2.60E+11** | **2.45E+11** | /mL plasma | FNTA | Miranda (2018) |
| **SGA** | 3rd | **2.60E+11** | **4.95E+11** | /mL plasma | FNTA | Miranda (2018) |
| **IUGR** | 1st | 1.09E+03 | 1.14E+03 | /µL plasma † | FC | Jadli (2017) |
|  | 3rd | 4.29E+02 | 1.82E+02 | /µL plasma † | FC | Bretelle (2003) |
|  |  | 2.23E+11 | 3.28E+11 | /mL plasma | NTA | Li (2020) |
| **PL** | 3rd | 1.09E+03 | 1.12E+03 | /µL plasma † | FC | Alijitos-Reig (2011) |
| **RM** | 3rd | 1.09E+03 | 1.30E+03 | /µL plasma † | FC | Alijitos-Reig (2011) |
| **UFL** | 3rd | 1.09E+03 | 9.36E+02 | /µL plasma † | FC | Alijitos-Reig (2011) |
| **SA** | 1st | **6.00E+11** | **5.50E+11** | /mL plasma | NTA | Monteiro (2020) |
| **PTB** | 1st | **5.02E+08** | **4.12E+08** | /mL plasma | FNTA | Menon (2020) |
|  | 2nd | **7.35E+08** | **6.89E+08** | /mL plasma | FNTA | Menon (2020) |
|  |  | 5.40E+09 | 7.48E+09 | /mL plasma | NTA | Truong (2017) |
|  | 3rd | **7.28E+08** | **6.82E+08** | /mL plasma | FNTA | Menon (2020) |
|  |  | **1.7E+11 (TNIL) 1.3E+11 (TIL)** | **1.85E+11** | /mL plasma | NTA | Menon (2019) |
|  |  | 4.12E+10 (TNIL) 4.64E+10 (TIL) | 4.15E+10 | /mL plasma | NTA | Tronco (2020) |
| **PPROM** | 3rd | **1.7E+11 (TNIL) 1.3E+11 (TIL)** | **1.70E+11** | /mL plasma | NTA | Menon (2019) |
|  |  | 4.12E+10 (TNIL) 4.64E+10 (TIL) | 4.60E+10 | /mL plasma | NTA | Tronco (2020) |
| **GDM** | 1st | 2.77E+11 | 1.27E+12 | /mL plasma | ELISA | Salomon (2016) |
|  | 2nd | 1.37E+12 | 2.85E+12 | /mL plasma | ELISA | Salomon (2016) |
|  |  | **1.20E+11** | **1.90E+11** | /mL plasma | NTA | James-Allan (2020) |
|  | 3rd | 2.38E+12 | 4.55E+12 | /mL plasma | ELISA | Salomon (2016) |
|  |  | 9.30E+06 | 7.40E+06 | /mL plasma | NTA | Zhang (2021) |
| **PE&FGR** | 3rd | 1.10E+03 | 1.08E+03 | /µL plasma † | FC | Alijitos-Reig (2012) |
|  | 3rd | 1.09E+03 | 2.83E+03 | /µL plasma † | FC | Jadli (2017) |
| **HIV** | 3rd | 6.16E+09 (>33weeks)  8.45E+09 (<34 weeks) | 9.96E+09 (>33weeks)  1.22E+10 (<34 weeks) | /mL plasma | ELISA | Pillay (2020) |
|  |  | **1.75E+04** | **2.25E+04** | /µL plasma † | FC | Moro (2016) |
| **HIV&EOPE** | 3rd | 6.16E+09 (>33weeks) | 3.71E+10 | /mL plasma | ELISA | Pillay (2020) |
| **HIV&LOPE** | 3rd | 8.45E+09 (<34 weeks) | 2.15E+10 (<34 weeks) | /mL plasma | ELISA | Pillay (2020) |
| **Malaria** | 3rd | **1.25E+04** | **1.50E+04** | /µL plasma † | FC | Moro (2016) |

1

st

**2.50E+03**

**4.90E+03**

2

nd

**2.95E+03**

**5.90E+03**

3

rd

**2.50E+03**

**5.90E+03**

/µL plasma †

FC

Campello (2018)

**APS**
